# SARS-Cov2 acute and post-active infection in the context of autoimmune and chronic inflammatory diseases

**DOI:** 10.1016/j.jtauto.2022.100154

**Published:** 2022-04-12

**Authors:** Regina Larionova, K. Byvaltsev, Оlga Kravtsova, Elena Takha, Sergei Petrov, Gevorg Kazarian, Anna Valeeva, Eduard Shuralev, Malik Mukminov, Yves Renaudineau, Marina Arleevskaya

**Affiliations:** a Central Research Laboratory, Kazan State Medical Academy, Kazan, Russia; b Institute of Fundamental Medicine and Biology, Kazan (Volga Region) Federal University, Kazan, Russia; c Institute of Fundamental Medicine, Kazan (Volga Region) Federal University, Kazan, Russia; d Institute of Environmental Sciences, Kazan (Volga Region) Federal University, Kazan, Russia; e Kazan State Academy of Veterinary Medicine Named After N.E. Bauman, Kazan, Russia; f Laboratory of Immunology, CHU Purpan Toulouse, INSERM U1291, CNRS U5051, University Toulouse III, Toulouse, France

**Keywords:** SARS-Cov2, Infection, Rheumatoid arthritis, Systemic lupus erythematosus, Risk factors, Inflammation, ACE2, angiotensin converting enzyme 2, ACPA, anti-cyclic citrullinated peptide autoAb, ANA, antinuclear autoAb, aPL, antiphospholipid, AutoAb, autoantibodies, BAFF/BlySS, B-cell-activating factor/B lymphocyte stimulator, CCL, chemokine ligand, COVID-19, coronavirus disease 2019, DMARDs, disease-modifying anti-rheumatic drugs, E, envelope, HEp-2, human epithelioma cell line 2, IFN-I, interferon type I, IFNAR, IFN-alpha receptors, Ig, immunoglobulin, IL, interleukin, IRF, interferon regulatory factor, ISGs, IFN-stimulated genes, ITP, immune-thrombocytopenic purpura, Jak, Janus kinase, LDH, lactate dehydrogenase, M, membrane, mAb, monoclonal Ab, MDA-5, melanoma differentiation-associated protein, MERS-Cov, Middle East respiratory syndrome coronavirus, MIS-C, multisystem inflammatory syndrome in children, N, nucleocapsid, NET, nuclear extracellular traps, NF-κB, nuclear factor-kappa B, NK, natural killer, NLRP3, NOD-like receptor family, pyrin domain containing 3, ORF, open reading frame, PACS, post-active COVID-19 syndrome, PAD-4, peptidylarginine deiminase 4, PAMPs, pathogen-associated molecular patterns, pDC, plasmacytoid dendritic cells, PMN, polymorphonuclear leukocytes, PRRs, pattern recognition receptors, RA, rheumatoid arthritis, RBD, receptor binding domain, RF, rheumatoid factor, RIG-I, retinoic acid-inducible gene I, ROS, reactive oxygen species, rt-PCR, reverse transcription polymerase chain reaction, S, spike, SAD, systemic autoimmune disease, SARS-Cov2, severe acute respiratory coronavirus 2, SjS, primary Sjögren's syndrome, SLE, systemic lupus erythematosus, SSc, systemic sclerosis, ssRNA, single-stranded ribonucleic acid, STAT, signal transducer and activator of transcription, TCR, T cell receptor, TLR, Toll-like receptor, TMPRSS2, transmembrane serine protease 2, TNF, tumor necrosis factor, Treg, regulatory T cells, VDJ, variable, diversity and joining Ig genes

## Abstract

The clinical and immunological spectrum of acute and post-active COVID-19 syndrome overlaps with criteria used to characterize autoimmune diseases such as rheumatoid arthritis (RA) and systemic lupus erythematosus (SLE). Indeed, following SARS-Cov2 infection, the innate immune response is altered with an initial delayed production of interferon type I (IFN–I), while the NF-kappa B and inflammasome pathways are activated. In lung and digestive tissues, an alternative and extrafollicular immune response against SARS-Cov2 takes place with, consequently, an altered humoral and memory T cell response leading to breakdown of tolerance with the emergence of autoantibodies. However, the risk of developing severe COVID-19 among SLE and RA patients did not exceed the general population except in those having pre-existing neutralizing autoantibodies against IFN-I. Treatment discontinuation rather than COVID-19 infection or vaccination increases the risk of developing flares. Last but not least, a limited number of case reports of individuals having developed SLE or RA following COVID-19 infection/vaccination have been reported. Altogether, the SARS-Cov2 pandemic represents an unique opportunity to investigate the dangerous interplay between the immune response against infectious agents and autoimmunity, and to better understand the triggering role of infection as a risk factor in autoimmune and chronic inflammatory disease development.

## Highlights

• SARS-Cov2 leads to a dysregulated immune response that mimics systemic autoimmune diseases.
• Sera from acute and post-active COVID-19 syndrome may contain SLE and RA associated autoantibodies.
• COVID-19 infection and vaccination impact on SLE and RA (incidence, flare) is limited.

## 1 SARS-Cov2 characteristics and infection

Our knowledge regarding SARS-Cov2 (severe acute respiratory coronavirus 2) infection, in COVID-19 (coronavirus disease 2019) patients, is largely based on research data obtained from SARS-Cov and MERS-Cov (Middle East respiratory syndrome coronavirus), two other representatives of the betacoronavirus family. This analogy can be done as the three human betacoronavirus share common characteristics: (i) similar genomes (80% homology between SARS-Cov2 and SARS-Cov; and 50% with MERS-Cov); (ii) an elevated rate and similar mode of transmission; and (iii) a severe clinical infection spectrum with lung damage and the development of a cytokine storm [[1], [2], [3]]. Of course, differences exist between them, which is beyond the scope of this review [4].

Members of the coronaviridae family share characteristics of being large, enveloped, and to possess a long ssRNA (single-stranded ribonucleic acid) genome, ranging from 25 to 32 kb (kilobases). The genome of coronaviridae contains four main structural proteins known as spike (S), envelope (E), membrane (M), and nucleocapsid (N) plus proteins implicated in RNA replication and non structural proteins that interfere with the host innate immune response [5,6]. Viral entry into host cells requires two steps, initially the RBD (receptor binding domain) present in the S1 part of the spike protein of SARS-Cov and SARS-Cov2 binds host cells using ACE2 (angiotensin converting enzyme 2) as a receptor. Next, the S2 part of spike is proteolytically activated for cellular fusion, which can be done at the S1/S2 boundaries by human proteases such as the TMPRSS2 (transmembrane serine protease 2), and by lysosomal cathepsins. In mature viruses, the three-dimensional structure of spike is also important with a spike protein present either in a “standing-up” position comprising the three receptor RBD/S1 heads sitting on the top of a trimeric structure, or else as a “lying-down” position for immune evasion [7]. ACE2 receptor expression presents an ubiquitous distribution with a high expression reported in epithelial cells of the respiratory (alveoli, mucous membrane of the oral cavity, nose, nasopharynx), digestive (stomach, intestines) and cardio-renal tracts, while a limited expression characterizes the brain and lymphoid tissues (lymph nodes, thymus, spleen, liver, and blood cells) [8] [5,9,10]. Accordingly, SARS-Cov2 can potentially infect all cell types, except those cells that do not express or express low amounts of ACE2 such as immune and red blood cells. Consequently, and since the mouth is the primary route of SARS-Cov2 infection and transmission, microbiological swabs from the nasopharynx are recommended to analyze the possible routes of viral transmission and infection [11].

ACE2 expression and activity are promoted by cigarette smoking, chronic obstructive pulmonary diseases, diabetes, heart diseases, and SLE (systemic lupus erythematosus), an autoimmune disease, and with the magnitude of response varying with sex and age. In SLE, the beginning of this phenomenon comes from a defective DNA methylation of the X chromosome in CD4^+^ T cells [12,13]. ACE2 serum concentrations are reported to be low in connective tissue diseases such as SLE, RA (rheumatoid arthritis), SjS (primary Sjögren's syndrome), and SSc (systemic sclerosis), while an elevated ACE2 level predicts the COVID-19 severity [14,15]. The presence of autoAb (autoantibodies) targeting ACE2 in SLE patients with vasculopathies has been reported [16]. Moreover, anti-TNFα (tumor necrosis factor) and Jak (Janus kinase)-inhibitors used in RA are effective for controlling ACE2 expression, which may contribute to prevention of infections and/or severe forms induced by COVID-19 [17,18].

Typically, the SARS-Cov2 viral load from upper respiratory tract samples (nasopharyngeal or throat swabs) peaks in the first week after the onset of infection and decreases rapidly over the next 2–4 weeks [19]. The mean duration of virus isolation from sputum samples (34 days) is longer than from nasopharynx (19 days). It's of further importance to note that viral load is positively correlated with severe acute respiratory symptoms and the viral clearance is delayed when age is over 65 years old [20]. In rare cases of viral pneumonia, viral ssRNA detection from oropharyngeal swabs remain positive for more than 4 months, usually in relation to a delayed or defective humoral immune response against SARS-Cov2 [21,22]. Moreover, viral RNA is also revealed in fecal samples but only in the mild cases of the infection [23]. After the first negative airway test, SARS-Cov2 can be detected in various organs, including intestines, kidney, heart, and brain several months after resolution of acute infection when using a rt-PCR (reverse transcription polymerase chain reaction) or a histological approaches [24,25]. Thus, its supports the concept that inflammation associated with SARS-Cov2 in reservoir organs contributes to PACS (post-active COVID-19 syndrome), which may be important in triggering autoimmune/inflammatory diseases [26,27].

## 2 Innate immune response against SARS-Cov2

**Fig. 1:**
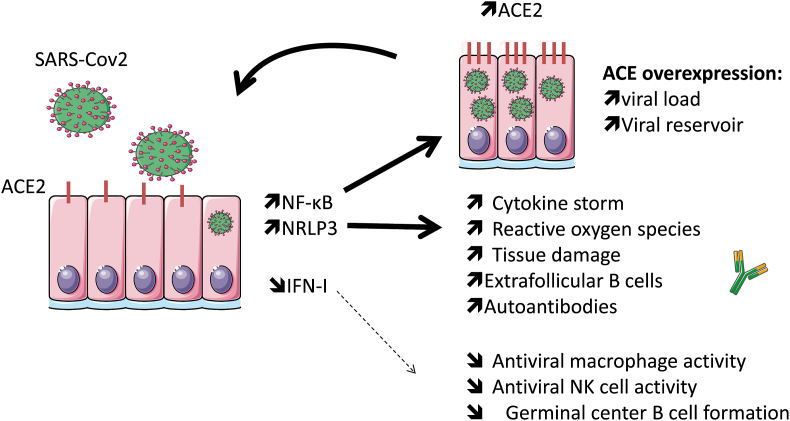
Following SARS-Cov2 infection, the initial immune response is altered with an activation of the NF-κB and inflammasome (NRLP3) pathways, while the production of interferon type I (IFN–I) is delayed. Activation of the NF-κB and inflammasome pathways lead to ACE2 (SARS-Cov2 receptor) overexpression that reinforces viral infection, and contributes to an exuberant inflammatory response known as a cytokine storm, at the onset of tissue damage, and the promotion of the autoreactive extrafolicular pathway. In contrast, IFN-I capacity to control the viral spread, innate and acquired immune response is affected.

Inhaled SARS-Cov2 particles infect nasal and alveolar epithelial cells, and a robust viral replication is promoted through a delayed production of IFN-I (interferon type I) leading enhancement of the cytopathic effect of the virus (Fig. 1). In addition, and through ACE2 binding and internalization in epithelial cells, SARS-Cov2 exerts a robust activation leading to the release of monocyte chemoattractant (e.g. CCL [chemokine ligand]-2, CCL-7) and pro-inflammatory cytokines (e.g. IL [interleukin]-6, IL-1β) [28,29]. Following their recruitment, circulating monocytes differentiate into pro-inflammatory macrophages through activation of the NF-κB (nuclear factor-kappa B) and inflammasome pathways that can lead, on one hand, to ACE2 overexpression to reinforce viral infection, and, on the other hand, to an exuberant inflammatory response known as a cytokine storm at the onset of tissue damage [28,30].

### 2.1 Delayed production of interferon type I

The critical event that determines the outcome of the disease is related to the capacity of SARS-Cov2 to delay the IFN-I/Jak-STAT (signal transducer and activator of transcription) response. This pathway is critical for controlling viral spread and possesses an immunomodulatory role on both the innate immune response through the control of macrophage phagocytosis and the NK (natural killer) cell response, and on the acquired immune response by acting on antigen-presenting cells for T cell activation, and on B cell maturation. Like other RNA viruses, viral proteins and the RNA component of the coronaviridae are detected by host sensors of PAMPs (pathogen-associated molecular patterns). Corresponding host sensors implicated in the IFN-I response and referred to as PRRs (pattern recognition receptors) include TLR (Toll-like receptor)-3 and TLR-4 located in the cell membrane, TLR-7 and TLR-8 located in endosomes, plus RIG-I (retinoic acid-inducible gene I) and MDA-5 (melanoma differentiation-associated protein) located in the cytoplasm [31]. Among them, RIG-I/MDA-5 and TLR-7/8 are the main sensors in SARS-Cov2 recognition leading to the recruitment and activation of the IRF (interferon regulatory factor)-3, IRF-5, and NF-kB p65, which are necessary for the IFN-I response. Next, IFN-I binds in an autocrine and paracrine response to the cell surface IFNAR (IFN-alpha receptors)-1/2 that trigger activation of the Jak-STAT signaling pathway, which in turn drives the expression of hundreds of ISGs (IFN-stimulated genes). In order to control the IFN-I/Jak-STAT pathway, several non-structural proteins of SARS-Cov2 are described as interactors of host proteins at the different steps of this pathway [32]. Moreover, the presence of neutralizing autoAb directed against IFN-I and/or genetic polymorphisms (TLR-3, TLR-7 and IRF-7) influencing the IFN-I/Jak-STAT pathway are reported in severe COVID-19 patients [33,[34], [35]].

**Fig. 2:**
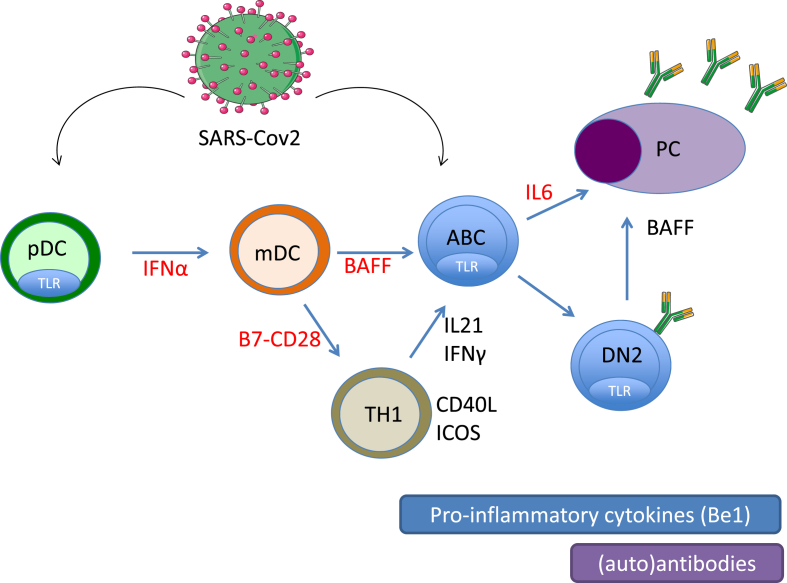
The extrafollicular pathway generates both viral antibodies and autoantibodies following infection with SARS-Cov2, and similar to autoimmune diseases this pathway is enhanced during severe COVID-19. The cellular actors include tissular plasmacytoid dendritic cells (pDC), monocytoid dendritic cells (mDC), T helper 1 cells (TH1) which can activate B cells into autoimmune B cells (ABC), double negative memory B cells (DN2) and autoreactive plasma cells (PC). The extrafollicular pathway is driven by interferon type I and II, and the cytokines: BAFF, IL-21 and IL-6. Therapeutic targets used in autoimmune diseases and COVID-19 are indicated in red. (For interpretation of the references to colour in this figure legend, the reader is referred to the Web version of this article.)

The presence of autoAb directed against IFN-I retrieved in SLE patients is suspected of being a risk for severe COVID19 [36]. This autoAb production results from an exacerbated IFN-I pathway that characterizes patients with systemic autoimmune diseases (SAD) [[37], [38], [39]], and the presence of neutralizing anti–IFN–I autoAb lower disease activity as reported in SLE but not in RA [40,41]. Moreover, polymorphisms causing activation of the IFN-I pathway result in a phenotype, known as interferonopathy, which recapitulates some of the manifestations of lupus [42]. Due to the key role played by the IFN-I pathway in the physiopathology of SLE, the control of the circulating levels of IFN-I represents an interesting therapeutic option in SLE that could be exploited by targeting pDC (plasmacytoid dendritic cells) that secrete inappropriate levels of IFN-I (e.g. anti-BDCA2/BIIB059), by targeting IFN-I or IFNAR, by controlling IFN-I production (e.g. hydroxychloroquine), and downstream by controlling autoAb production and the formation of immune complexes with apoptotic nucleic acids (e.g. Belimumab) via the cytokine BAFF/BlySS (B-cell-activating factor/B lymphocyte stimulator), which contributes in an amplification loop to pDC hyperactivation [[43], [44], [45], [46]] (Fig. 2). The use of mAb (monoclonal Ab) to target IFN-I or IFNAR in SLE patients influences the innate immune response with a higher report of upper respiratory tract infections, nasopharynx, bronchitis and herpes reactivation [47].

### 2.2 Activation of the NF-kappaB pathway

The inhibitory effect of SARS-Cov 2 on the IFN-I pathway is counterbalanced by a hijacking of the NF-κB pathway, which is responsible for upregulating the expression of inflammatory cytokines (e.g. IL-1β, IL-8, IL-6), chemokines (e.g. CXCL-10, CCL2), alarmins and inducible enzymes, which paves the pathway for a cytokine storm, ACE2 overexpression (see above), attraction of immune cells to the inflammatory sites, and as a consequence, organ damage [6,48,49]. The hijacked effect can be massive as seen in post-mortem investigations of deaths from COVID-19. An immune redistribution is reported that consists of the presence in tissues macrophages and monocytes supplemented with PMN (polymorphonuclear leukocytes), eosinophils and CD4^+^ T cells, while spleen and lymph nodes are decimated due to apoptosis in lymphoid organs [15,50]. The inappropriately high blood level of proinflammatory cytokines is associated with lymphopenia, disease severity and higher morbidity. However, authors differ on biomarkers of poor prognosis: IL-6 and IL-10 [51], IL-2R, IL-6, IL-10, TNF-α [52]; IL-1B, IFN-γ, IFN-inducible protein 10, and monocyte chemoattractant protein 1 [53]); and CXCL-10, CCL2 and proteins encoded by IFN-stimulated genes [6].

### 2.3 Activation of the NRP3 inflammasome

SARS-Cov2 triggers NLRP3 (NOD-like receptor family, pyrin domain containing 3) inflammasome priming directly through viral protein (e.g., ORF [open reading frame]-3, envelope, nucleocapsid) and indirectly in response to various signals that include but are not limited to ion fluxing, protein aggregation, the NF-κB pathway, and ROS (reactive oxygen species). When assembled, the inflammasome starts to cleave and release the proinflammatory cytokines IL-1β and IL-18 into their mature forms and gasdermin D into its active fragment necessary to initiate the programmed cell death pyroptosis process [28]. A moderate NLRP3 inflammasome activation is beneficial due to its capacity to eliminate microbial infection, to repair damaged tissues, and to induce T and B cell responses, but when the activation becomes major this can lead to an excessive inflammation, tissue damage and pain. In COVID-19 patients, disease severity and mortality are correlated with IL-1β and IL-18 levels and the release of LDH (lactate dehydrogenase) by pyroptosis.

The NLRP3 inflammasome is overactivated in multiple autoinflammatory disorders including SLE and RA [54]. In addition, NLRP3 inflammasome related polymorphisms are associated with susceptibility, disease severity and/or therapeutic responses in SAD [55], and they are suspected of contributing to disease severity in COVID-19 [56].

## 3 Acquired immune response

### 3.1 Humoral response

Anti-SARS-Cov2 seroconversion starts with a median of 9–21 days from onset of symptoms, and responsiveness broadens with participation of the three Ig (immunoglobulin) isotypes IgM/IgG/IgA [57]. The most commonly produced Ab are those targeting the Spike glycoprotein and the highly conserved nucleocapsid protein. Neutralizing anti-SARS-Cov2 Ab are directed against the RBD/S1 domain and prevent the interaction between the coronavirus and the cellular receptors, Spike and ACE2, respectively. Regarding IgG neutralizing anti-spike Ab, the detection starts very early (at 5 days post-infection in severe COVID-19), the titer peaks at 15–20 days and with higher levels reported in severe COVID-19, reaching a plateau that starts to decline from 40 days post-onset, and next a rapid decline is reported [[58], [59], [60]]. Qualitatively, the humoral response evolved into an IgG1 dominated response with a limited VDJ (variable, diversity and joining Ig genes) segment recombination and autoreactivity (e.g. VH4-34 9G4 idiotope), which is consistent with an extrafollicular B cell response.

Plasma cell development is responsible for the production of protective anti-infectious Ab and traditionally this arises in secondary lymphoid organs through germinal center responses that control somatic hypermutation, class switching and autoreactivity. However, early production of elevated levels of neutralizing anti-SARS-Cov2 Ab can also occur from extrafollicular locations, a pathway chronically mobilized in SLE, and RA [60]. Driven by the IFN-I pathway, the extrafollicular B cell response is elevated in patients with severe COVID-19 [61]. Extrafollicular B cells and plasmablasts have been reported in the thoracic, cervical, mediastinal and hilar lymph nodes missing germinal centers from severe COVID-19 patients, peripheral blood from SLE, and in synovium from RA patients [62,63].

### 3.2 T cell response

A common symptom in COVID-19 is lymphopenia with a reduction in the absolute number of CD4^+^ T cells, in particular ones expression IFN-γ, as well as CD8^+^ T cells and B cells [52,64,65]. Lymphopenia gradually decreased as the severity of the infection increased, showing a negative correlation with the proinflammatory cytokine levels. Moreover, T cells presented reduced diversity in their TCR (T cell receptor) usage and a functional exhaustion was demonstrated in the severe infection cases [66].

When regarding SARS-Cov/SARS-Cov2 CD4^+^ and CD8^+^ T cells, the memory T cell response is maintained for at least 4–6 years after infection in 70%–100% of patients, and is partially correlated with the infection severity [[67], [68], [69], [70]]. The T cell epitopes are more widely represented than those of B cells [71]. Study of mouse models demonstrated that T cells alone may control SARS-Cov and depletion of CD4 T cells during SARS-Cov infection resulted in impaired viral clearance and reduced neutralizing Ab titers [72,73]. Effector CD4^+^ T cells express IFN-γ and other cytokines, while CD8^+^ T cells producing IL-10 and Treg (regulatory T cells) protect against an excessive immune response [30].

## 4 Tolerance breakdown

### 4.1 Autoantibodies

An elevated prevalence of autoAb is often reported in patients with acute COVID-19, and the most frequent associations are described with aPL (antiphospholipid autoAb), ANA (antinuclear autoAb), RF (rheumatoid factor), and ACPA (anti-cyclic citrullinated peptide autoAb) [74]. Among patients hospitalized with COVID-19, more than half presented aPL autoAb including a positive lupus anticoagulant assay, anti-cardiolipin and/or anti-β2GPIs autoAbs predominantly of the IgM isotype [75,76]. However, no association between aPL autoAb and thromboembolism outcomes were reported as explained by the analysis of the aPL epitopes recognized during COVID-19, which are outside the pathogenic epitopes retrieved in aPL syndrome [[77], [78], [79]]. The prevalence of ANA tested on HEp-2 (human epithelioma cell line 2) by immunofluorescence range from 30 to 50% in hospitalized patients with COVID-19 with the particularity to be weakly positive, to present a cytoplasmic rather than a nuclear pattern and, when related to SAD-associated anti-nuclear autoAb, a single nuclear target is reported [80,81]. Regarding the relationship between severe COVID-19 and RA-associated autoAb, IgM RF is primarily present (20–60%) and few cases of ACPA are reported [[82], [83]]. Such observations are not restricted to SARS-Cov2 infection as the level of natural and low affinity autoAb is typically retrieved during various infections and returns to normal when the infectious inflammation subsides [84,85]. Taken together, this implies that autoAb production associated with COVID-19 infection is predominantly associated with an immune system activation rather than driven by a specific SARS-Cov2 specific immunopathological process. However, based on the implication of infections as a risk–factor for autoimmune diseases [86], it could not be excluded that COVID-19 infection or vaccine can disturb self-tolerance and trigger an autoimmune response. This is reported with both ITP (immune-thrombocytopenic purpura) and MIS-C (multisystem inflammatory syndrome in children), a pediatric autoimmune medium-vessel vasculitis [[87], [88], [89], [90]]. Autoimmunity induced by COVID-19 can be promoted through cross-reactivity with foreign antigens (molecular mimicry), or through direct activation by the emergence of new antigenic epitopes as a result of tissue damage (e.g. nuclear apoptotic antigens) and/or post-translational modifications of self-proteins (e.g., citrullination).

### 4.2 Molecular mimicry

Molecular mimicry between COVID-19 viral epitopes and auto-epitope leading to autoAb production was initially suspected based on the use of bioinformatic models with linear sequence homology models, but with limited, if any, experimental evidence [91]. Indeed, cross-mimicry was not confirmed through competition experiments when using increased amounts of the autoantigen to test reactivity against SARS-Cov2, or vice versa when testing autoreactivity from a large panel of sera with organ and non-organ-specific autoimmune diseases in the presence SARS-Cov2 [92].

### 4.3 Post-translational modifications (new epitops)

An elevated citrullination process indicative of neutrophil extracellular trap formation, in response to neutrophil activation/NETosis (neutrophil extracellular trap releases), and PAD-4 (peptidylarginine deiminase 4) overexpression is reported in the lung from severe COVID-19 [93,94]. Citrullinated Histone-3 in COVID-19 was further shown to be associated with inflammation and neutrophil count [95]. The use of peripheral blood citrullinated nucleosomes levels as a biomarker has been proposed to follow severe COVID-19 [96]. As a consequence, long term exposure to citrullinated proteins may lead to the formation of ACPA and/or anti-chromatin/nucleosome autoAb that characterize RA and SLE, respectively. This is in line with a recent report describing RA in a case of chronic-COVID-19 with ACPA positivity [97].

## 5 SARS-COV-2/Covid19 vaccination and autoimmune and chronic inflammatory diseases: friends, foes or both

Based on the previous arguments that COVID-19 shares similarities with SLE and/or RA in terms of clinical manifestations, therapeutic management, pathogenic mechanisms and immune responses one may argue: (i) that patients with SAD are at higher risk of developing severe COVID-19; (ii) that drugs used to treat SAD control or exacerbate the inappropriate immune response to SARS-Cov2; (iii) that SAD severity increases following COVID-19 vaccination and/or exposure to SARS-Cov2 as reported with other respiratory viruses; and (iv) that PACS predispose to the emergence of SAD.

### 5.1 SAD, medications and severe COVID19 risk

Considering the pathological crosstalk between COVID-19 and SAD, the risk of developing COVID-19 and exacerbating COVID-19 outcomes was first suspected in SLE and RA patients. However, data are now accumulating that counter this hypothesis since COVID-19 incidence in SLE and RA did not exceed that in the general population, and the hospitalization rate appears similar to that identified in the general population. However, such an association needs to be better understood since lupus nephritis represents a predictive risk factor of severe COVID-19 and a poor onset prognosis with long-term COVID-19 is also observed among SLE patients [[98], [99], [100], [101], [102]].

The use of DMARDs (disease-modifying anti-rheumatic drugs) in SLE and RA elicited a substantial effect on the innate and acquired immune response, supporting the idea that DMARDs used prior to SARS-Cov2 infection can influence COVID-19 outcome with three groups of responses [103,104]. First, RA patients receiving anti-TNFα agents are at lower risk of hospitalization and death [105]. Second, glucocorticoids (>10 mg/day), DMARDs when used in combinations, and the anti-B cell mAb rituximab increase the risk of COVID-19 outcomes in RA patients [106]. Third, patients treated with (hydroxy)chloroquine, conventional DMARDs in monotherapy, T cell co-stimulatory signal inhibitors (e.g. abatacept), IL-6 inhibitors, and non-steroidal anti-inflammatory drugs presented a similar occurrence and outcome of COVID-19 as observed with the controls. Regarding hydroxychloroquine, the risk of adverse reactions increases when doses are higher than usual in hospitalized COVID-19 patients [107]. Regarding Jak-inhibitors, conflicting results have been reported, which may be related to the dual effect of Jak-inhibitors in controlling both the innate immune response and the cytokine storm associated with severe COVID-19 [108].

The presence of pre-existing neutralizing autoAb against IFN-I, first described in SLE and subsequently in patients treated with IFN (alpha or beta) has been demonstrated to account for at least 10–20% of severe COVID19 with pneumonia the anti–IFN–I autoAb are absent from asymptomatic/mild COVID-19 [33]. The prevalence increases in patients over 65 years old and predominates in men. Except for COVID-19, the presence of these neutralizing autoAb is generally considered to be clinically silent.

### 5.2 SAD activity in response to viral infections and vaccinations

Lessons from seasonal influenza infection in patients with SLE have established influenza infections as a trigger for SLE flares, through a suspected exacerbation of the IFN-I/Jak-STAT signaling pathway [[109], [110], [111]]. In RA, complications evolved with the seasonal waves of influenza [112]. We have further reported in RA patients that systemic herpes reactivation is associated with an exacerbation of disease activity [113,114], and that upper respiratory tract infections precede RA at the preclinical stage in a genetically predisposed population [115].

SLE flare risk was evaluated after SARS-Cov2 infection revealing that treatment discontinuation rather than COVID-19, usually mild, is at the origin of disease flares [101]. As a consequence, drug withdrawal should be avoided or evaluated with caution on a case-by-case basis including during pregnancy [116]. When considering the risk of a flare following COVID-19 vaccination in SLE patients, the risk remains similar in pre- and post-vaccination [117,118].

### 5.3 SAD risk after COVID19 infection

#### 5.3.1 Clinical homology

Acute and PACS are characterized by a large panel of clinical manifestations ranging from asymptomatic symptoms to fatal respiratory failure, and are often associated with manifestations shared with SAD such as musculoskeletal, dermatological, pulmonary, digestive, cardiovascular, kidney injury, thromboembolic events and neurological symptoms [88,91,97,[119], [120], [121], [122], [123], [124], [125], [126], [127], [128], [129], [130]]. Potential mechanisms contributing to SAD-associated clinical symptoms include direct virus replication in acute/chronic infection, inflammation, and immune changes in response to the infection [131]. In pediatric forms, overlapping features have been noted with Kawasaki disease, a pediatric autoimmune medium-vessel vasculitis, such as coronary artery dilation or aneurysm, fever, gastrointestinal symptoms, skin rash, mucocutaneous lesions, toxic-shock syndrome, and neurological symptoms [90].

#### 5.3.2 COVID19 and SLE

SLE starts with an inappropriate innate and acquired immune response to nucleic-acid-containing apoptotic cellular components triggered by sustained production of IFN-I [132]. In this process, candidate environmental risk factors include UV light exposure and chronic virus exposition such as cytomegaloviruses (CMV), Epstein Barr viruses (EBV), parvovirus B19 and retroviruses. SARS-Cov2 was recently added to this list as ANA and aPL auto-Ab together with an IFN-I signature and inappropriate immune system activation characterize acute COVID-19 and PACS (see above). However and based on the low number of reports of SLE confirmed to be triggered by SARS-Cov2, the SLE-like immune profile associated with COVID-19 is not sufficient to trigger SLE, then SARS-Cov2 infection represents an ideal experimental model for understanding some aspects of SLE pathophysiology and to prevent the emergence of SLE in COVID-19 infected individuals.

#### 5.3.3 COVID19 and RA pathophysiology

RA development occurs in genetically predisposed individuals exposed to environmental factors, which ultimately result in an inflammatory destructive synovial response [133]. As reported with tobacco smoking and air pollutants, COVID-19 can act on cells in mucosal sites (mouth, lung and gut) to promote the induction of PADI-4 and in turn to the formation of citrullinated histones [134,135]. This process is suspected of becoming chronic in PACS since lung sensory neurons and the gut represent mucosal reservoirs for SARS-Cov2 [26,136,137]. Moreover, COVID-19 infection is primarily a respiratory infection with 70–80% of patients presenting radiographic lung involvement that appear simultaneously with fever, in some cases, even precedes fever [138,139]. COVID-19 chest computed tomography images such as ground glass opacities may resemble rheumatologic SAD including SLE and RA with extensive lung involvement, making the diagnosis of viral infection challenging [140]. Due to inflammation, bone erosion leading to rheumatoid nodules may be detected [141], and viral SARS-Cov2 RNA retrieved in the synovial fluid [142,143]. The shift from reactive arthritis following COVID-19 infection to RA remains exceptional, which supports the possibility that additional genetic and environmental factors are required [144].

## 6 Conclusion

The new SARS-Cov2 infection that paralyzed the world presented a unique opportunity to investigate the unsolved problem of the interaction between an anti-infectious immune response and autoimmunity. For that, several key questions remain to be answered including (i) the role played by transitory (e.g. vaccine) and chronic exposition; (ii) the location of the viral reservoir and its accessibility to the immune system; (iii) the capacity of SARS-Cov2 to initiate an autoreactive program; (iv) the predisposition and protective factors implicated in this shift; and (v) the characteristics of immune cells involved.

## Funding

This study was supported by research funding from the “Russian science foundation” (No17-15-01099).

## Declaration of competing interest

None.
